# The C-reactive protein/albumin ratio predicts overall survival of patients with advanced pancreatic cancer

**DOI:** 10.1007/s13277-016-5122-y

**Published:** 2016-06-25

**Authors:** Mengwan Wu, Jing Guo, Lihong Guo, Qiang Zuo

**Affiliations:** Department of Oncology, Nanfang Hospital, Southern Medical University, 1838 North Guangzhou Avenue, Guangzhou, Guangdong Province 510515 China

**Keywords:** Advanced pancreatic cancer, Inflammation, CRP/Alb ratio, Prognostic score, Survival

## Abstract

Recent studies have demonstrated the prognostic value of the C-reactive protein/albumin (CRP/Alb) ratio in cancer. However, the role of the CRP/Alb ratio in advanced pancreatic cancer (PC) has not been examined. A retrospective study of 233 patients with advanced PC was conducted. We investigated the relationship between the CRP/Alb ratio, clinicopathological variables, and overall survival (OS). The optimal cutoff point of the CRP/Alb ratio was 0.54. A higher CRP/Alb ratio was significantly associated with an elevated neutrophil-lymphocyte ratio (NLR) (*P* < 0.001) and higher modified Glasgow prognostic score (mGPS) (*P* < 0.001). Using univariate analyses, we found that the age (*P* = 0.009), disease stage (*P* < 0.001), NLR (*P* < 0.001), mGPS (*P* < 0.001), and CRP/Alb ratio (*P* < 0.001) were significant predictors of OS. Patients with a higher CRP/Alb ratio had a worse OS than patients with a lower CRP/Alb ratio (hazard ratio (HR) 3.619; 95 % CI 2.681–4.886; *P* < 0.001). However, the CRP/Alb ratio was identified as the only inflammation-based parameter with an independent prognostic ability in the multivariate analyses (*P* < 0.001). The pretreatment CRP/Alb ratio is a superior prognostic and therapeutic predictor of OS in advanced PC.

## Introduction

Pancreatic cancer ranks as the 12th most commonly diagnosed cancer and seventh leading cause of cancer death; there are an estimated 337,900 new cases and 330,400 deaths occurring in 2012 worldwide [1]. Most patients are diagnosed at an advanced stage and have a rapid clinical decline, culminating in death within less than 1 year after diagnosis. The high mortality is associated with a limited chance of curative resection at the time of diagnosis, as surgical resection offers the only prospect of long-term survival or cure. Moreover, one study showed that the median survival in patients with curative resection was 14.9 months longer than in those with an advanced stage (19.1 vs 4.2 months). Similarly, the 5-year survival rates were higher in the operable patients (19.3 vs 0.8 %) [2]. Although surgical resection has improved the prognosis of pancreatic cancer patients, it continues to have a poor prognosis.

Currently, although continuous advances in modern diagnostic imaging have been achieved, most established prognostic factors continue to rely on surgical exploration, such as the tumor size, histologic grade, and vascular and nodal involvement. Many patients at an advanced stage also undergo a morbid operative procedure during the course of pancreatic lesion evaluation. Therefore, it is important to identify some easily obtainable and reliable prognostic factors for better risk stratification and optimal treatment plans. With the increasing number of studies suggesting that tumor-elicited inflammation plays a key role in malignant transform and tumor progression [3–6], some inflammation-based prognostic factors have been explored in many cancers during the course from bench to bedside. These factors are all easily available from peripheral blood samples, including the neutrophil-lymphocyte ratio (NLR), platelet-lymphocyte ratio (PLR), white cell count and C-reactive protein (CRP) combined into the prognostic index (PI), albumin and lymphocyte count in the prognostic nutritional index (PNI), and CRP- and albumin-based factors of the modified Glasgow prognostic score (mGPS) and C-reactive protein/albumin (CRP/Alb) ratio [7–12]. Among these indicators, the CRP/Alb ratio has been reported as a novel reliable marker in different cancers such as lung, liver, gastric, and esophageal cancer [8, 13–16]. However, the role of the CRP/Alb ratio in patients with advanced pancreatic cancer has not previously been elucidated.

In this retrospective study, we examined the prognostic value of the CRP/Alb ratio and compared it to other established inflammation-based prognostic scores.

## Materials and methods

### Patients

We enrolled 233 patients who were diagnosed with advanced pancreatic cancer in Nanfang Hospital of Southern Medical University (Guangzhou, Guangdong Province, China) between January 2011 and December 2014. All medical records were retrospectively reviewed. The following inclusion criteria were used: (1) cytologically or histologically diagnosed as pancreatic adenocarcinoma; (2) survival of at least 30 days after diagnosis; (3) confirmed stage III or IV based on the AJCC/UICC TNM staging system (the 7th edition); and (4) pretreatment laboratory data were available. Patients suffering from detectable acute inflammation were excluded. Selected patients were carefully followed up after pathological diagnosis until September 30, 2015, or death from any cause.

### Clinical data collection

Baseline characteristics were obtained from the electronic medical record system, including the age; gender; tumor location and stage; pretreatment laboratory counts of white cells, neutrophils, lymphocytes, and platelets; tumor markers (carcinoembryonic antigen 19-9, CA19-9, and carcinoembryonic antigen (CEA)); levels of CRP and albumin; and therapeutic information. Based on previous studies, the CRP/Alb ratio was calculated by dividing the serum CRP level by the serum albumin level [17]. The mGPS combined the CRP and albumin concentrations. Patients who had both elevated CRP (>1 mg/dl) and decreased albumin levels (<3.5 g/dl) were assigned a score of 2. Patients with only elevated CRP (>1 mg/dl) were assigned a score of 1, and patients with neither of these abnormalities were assigned a score of 0 [18]. NLR and PLR were both categorized into two groups according to the cutoff values of >5 and >150, respectively [19, 20].

### Statistical analyses

Comparisons between groups were performed using the chi-square test. The optimal cutoff value of the CRP/Alb ratio was determined using a web-based system, R software-engineered, which was designed by Budczies J et al. (http://molpath.charite.de/cutoff/) [21]. The overall survival (OS) was measured from the day of pathological diagnosis to death from any cause or the final date of follow-up. The OS curves were generated using the Kaplan–Meier method, and differences between groups were compared using the log-rank test. Prognostic variables that were significant in univariate analyses were selected for multivariate Cox proportional hazard model analyses using the forward stepwise method. Statistical analyses were performed with SPSS 21.0 (IBM Corporation, Armonk, NY, USA). A two-sided *P* value <0.05 was considered statistically significant.

## Results

### Patient characteristics

A total of 233 patients who were diagnosed with advanced pancreatic cancer in Nanfang Hospital were evaluated. They had a median age of 62 years (range 26–85). Among these patients, 156 (67.0 %) were males and 77 (33.0 %) were females, while the numbers of patients with locally advanced and metastasis disease were 83 (35.6 %) and 150 (64.4 %), respectively. Nearly half of patients had a primary pancreatic head tumor (*n* = 104, 44.6 %). The pretreatment blood sample analyses showed that the median CA19-9 level for the entire group was 427 U/ml, while the CEA was a median of 3.45 ng/ml. More than two thirds of patients received gemcitabine monotherapy as first-line treatment (*n* = 162, 69.5 %). The baseline characteristics of the 233 patients are shown in Table 1.

**Table 1:** Clinicopathological characteristics of patients with pancreatic cancer (*n* = 233)

Factor	Number (%)
Age (years)	
<62	111 (47.6)
≥62	122 (52.4)
Gender	
Female	77 (33.0)
Male	156 (67.0)
Disease stage	
Locally advanced	83 (35.6)
Metastasis	150 (64.4)
Location	
Head	104 (44.6)
Body	64 (27.5)
Tail	37 (15.9)
Diffusion	28 (12.0)
CRP/Alb	
<0.54	159 (68.2)
≥0.54	74 (31.8)
NLR	
<5	176 (75.5)
≥5	57 (24.5)
PLR	
<150	115 (49.4)
≥150	118 (50.6)
mGPS	
0	119 (51.1)
1	76 (32.6)
2	38 (16.3)
CA19-9	
<Median (427 U/ml)	116 (49.4)
>Median	117 (50.6)
CEA	
<Median (3.46 ng/ml)	116 (49.4)
>Median	117 (50.6)
Treatment	
No chemotherapy	71 (30.5)
Chemotherapy	162 (69.5)
End-point	
Alive	6 (2.6)
Dead	227 (97.4)

*CRP*/*Alb* C-reactive protein/albumin, *NLR* neutrophil-lymphocyte ratio, *PLR* platelet/lymphocyte ratio, *mGPS* the modified Glasgow prognostic score, *CA19*-*9* carbohydrate antigen 19-9, *CEA* carcinoembryonic antigen

### Cutoff point determination for the CRP/Alb ratio

**Fig. 1:**
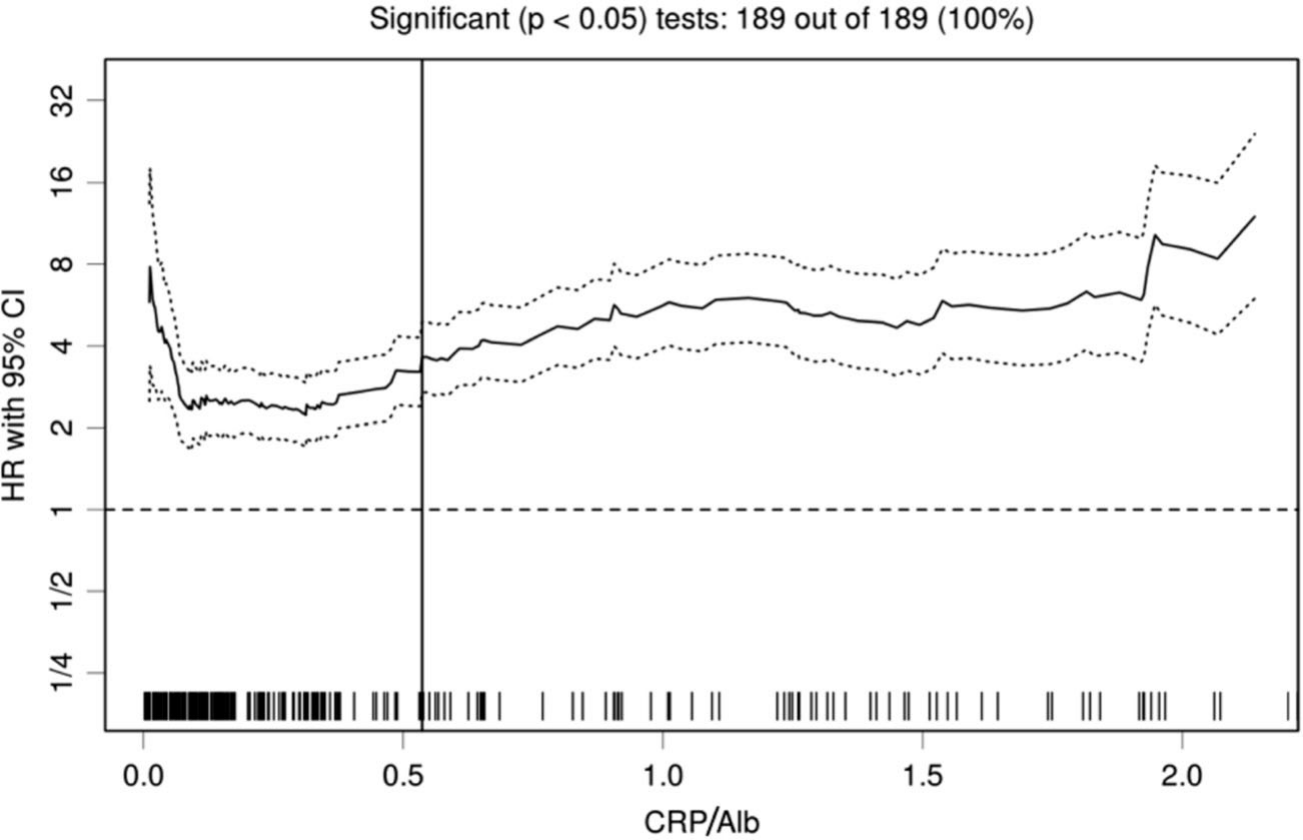
The hazard ratio (HR) for progression-free survival according to the cutoff points of the CRP/Alb ratio in patients with advanced PC. A *vertical line* designates the chosen cutoff point. The plots were generated using Cutoff Finder

The median value of the CRP/Alb ratio was 0.241 (range, 0.002–6.728). The analyses that were performed with the biostatistical tool Cutoff Finder showed that a wide range of cutoff points for the CRP/Alb ratio were significant (Fig. 1). For overall survival, the optimal cutoff point of the CRP/Alb ratio for stratifying patients with advanced pancreatic cancer (PC) was 0.54. Based on this result, all patients were categorized into the CRP/Alb-high (*n* = 74, 31.8 %) and CRP/Alb-low (*n* = 159, 68.2 %) groups.

### Correlation of the CRP/Alb ratio with clinicopathological parameters

The relationships between the CRP/Alb ratio and other variables are shown in Table 2. A higher CRP/Alb ratio was significantly associated with an elevated NLR (*P* < 0.001) and higher mGPS score (*P* < 0.001). In addition, fewer patients in the high CRP/Alb ratio group received chemotherapy (*P* < 0.001) compared to the low CRP/Alb ratio group. However, there were no significant differences in the age (*P* = 0.943), gender (*P* = 0.103), disease stage (*P* = 0.200), tumor location (*P* = 0.084), PLR values (*P* = 0.242), CA19-9 levels (*P* = 0.100), and CEA levels (*P* = 0.100) between the CRP/Alb-high and CRP/Alb-low groups.

**Table 2:** Correlation of the CRP/Alb ratio and clinicopathological characteristics of PC patients

Characteristics	CRP/Alb ratio <0.54, *n* (%)	CRP/Alb ratio ≥0.54 *n* (%)	*P* value
Age			0.943
<62	76 (47.8)	35 (47.3)	
≥62	83 (52.2)	39 (52.7)	
Gender			0.103
Female	58 (36.5)	19 (25.7)	
Male	101 (63.5)	55 (74.3)	
Disease stage			0.200
Locally advanced	61 (38.4)	22 (29.7)	
Metastasis	98 (61.6)	52 (70.3)	
Location			0.084
Head	67 (42.1)	37 (50.0)	
Body	43 (27.1)	21 (28.4)	
Tail	24 (15.1)	13 (17.6)	
Diffusion	25 (15.7)	3 (4.0)	
NLR			0.000*
<5	137 (86.2)	39 (52.7)	
≥5	22 (13.8)	35 (47.3)	
PLR			0.242
<150	84 (52.8)	33 (44.6)	
≥150	75 (47.2)	41 (55.4)	
mGPS			0.000*
0	119 (74.8)	0 (0)	
1	34 (21.4)	42 (56.8)	
2	6 (3.8)	32 (43.2)	
CA19-9			0.100
<Median (427 U/ml)	85 (53.5)	31 (41.9)	
>Median	74 (46.5)	43 (58.1)	
CEA			0.100
<Median (3.46 ng/ml)	85 (53.5)	31 (41.9)	
>Median	74 (46.5)	43 (58.1)	
Treatment			0.049*
No chemotherapy	42 (26.4)	29 (39.2)	
Chemotherapy	117 (73.6)	45 (60.8)	

* Significant differences between patients with the CRP/Alb < 0.54 and patients with the CRP/Alb ≥ 0.54

### Association between the CRP/Alb ratio and OS

**Fig. 2:**
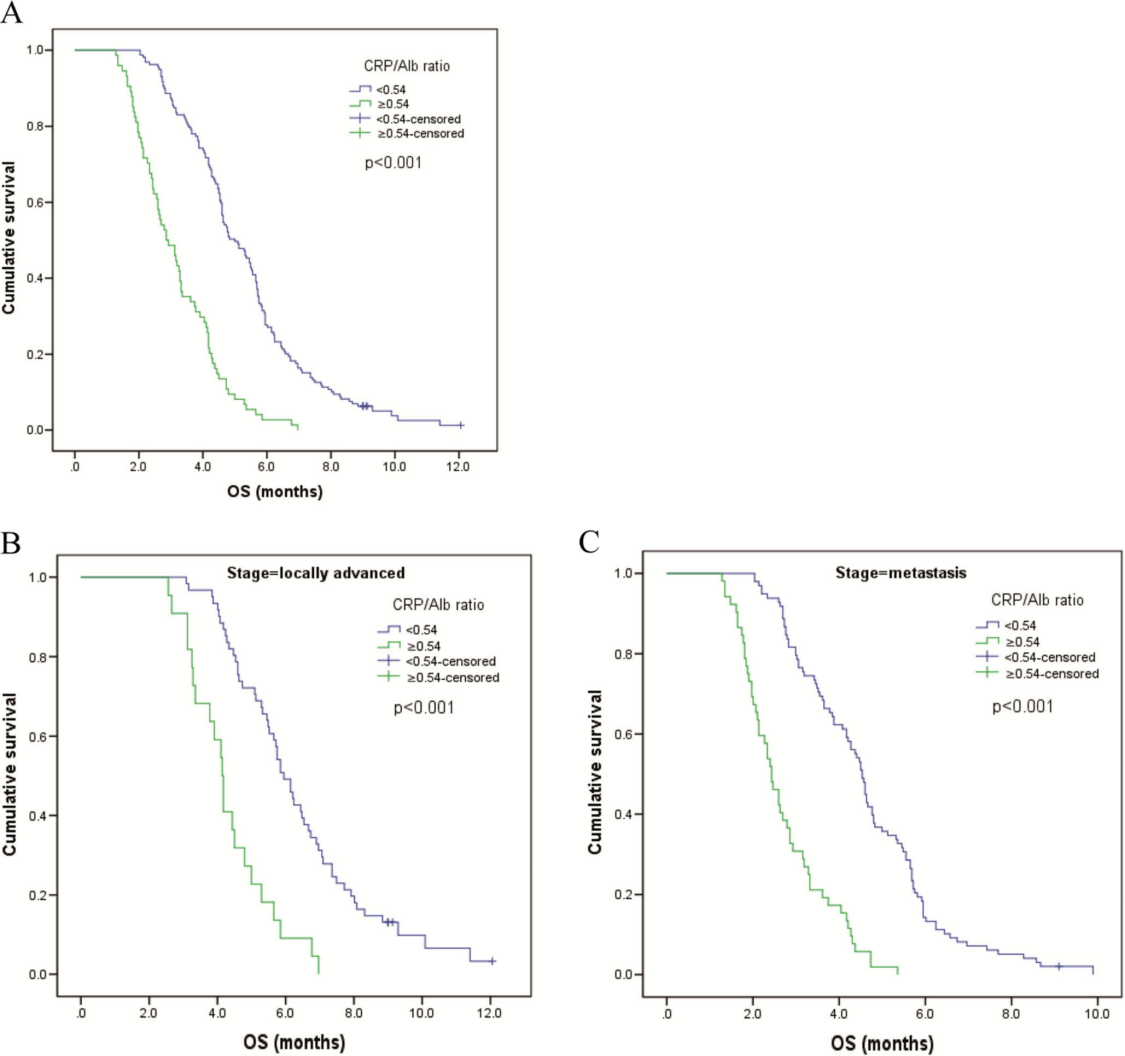
Kaplan–Meier survival curves showing the difference between the high CRP/Alb ratio and low CRP/Alb ratio groups **a** in all patients, **b** in patients with locally advanced disease, and **c** in patients with metastatic disease

The results of the univariate and multivariate analyses are presented in Table 3. The median survival time of all of the patients was 4.4 months (95 % CI 4.16–4.58 months), and 227 (97.4 %) patients died by their final follow-up visit. Based on the cutoff point of the CRP/Alb ratio, patients were divided into two groups (<0.54, *n* = 159 and ≥0.54, *n* = 74). In the univariate analyses of survival, the low CRP/Alb ratio group had a longer median overall survival than the high CRP/Alb ratio group (5.0 vs 2.9 months, *P* < 0.001). Additionally, when patients were stratified by disease stage, the mortality rate of patients with increased C-reactive protein/albumin ratio (CAR) was also higher in both the locally advanced subgroup (5.9 vs 4.1 months, *P* < 0.001) and metastasis subgroup (4.5 vs 2.4 months, *P* < 0.001) (Fig. 2).

**Table 3:** Prognostic factors for overall survival identified by univariate and multivariate analyses

	Univariate analysis		Multivariate analysis	
	HR (95 % CI)	*P* value	HR (95 % CI)	*P* value
Age (years)				
<62	Ref		Ref	
≥62	1.419 (1.089–1.850)	0.009*	1.681 (1.280–2.209)	0.000*
Gender				
Female	Ref			
Male	1.099 (0.834–1.449)	0.501		
Disease stage				
Locally advanced	Ref		Ref	
Metastasis	2.317 (1.745–3.076)	0.000*	2.620 (1.939–3.540)	0.000*
Location				
Head	Ref			
Body	0.846 (0.553–1.295)	0.441		
Tail	0.894 (0.567–1.408)	0.627		
Diffusion	0.962 (0.582–1.590)	0.880		
CRP/Alb				
<0.54	Ref		Ref	
≥0.54	3.619 (2.681–4.886)	0.000*	3.995 (2.644–6.034)	0.000*
NLR				
<5	Ref		Ref	
≥5	2.564 (1.870–3.517)	0.000*	1.049 (0.734–1.499)	0.795
PLR				
<150	Ref			
≥150	1.260 (0.969–1.639)	0.085		
mGPS				
0	Ref		Ref	
1 + 2	2.369 (1.806–3.109)	0.000*	1.226 (0.829–1.812)	0.308
CA19-9				
<Median (427 U/ml)	Ref			
>Median	1.104 (0.849–1.436)	0.459		
CEA				
<Median (3.46 ng/ml)	Ref			
>Median	1.298 (0.998–1.688)	0.052		
Treatment				
No chemotherapy	Ref		Ref	
Chemotherapy	0.146 (0.103–0.205)	0.000*	0.174 (0.121–0.250)	0.000*

* Significant differences between patients with the CRP/Alb < 0.54 and patients with the CRP/Alb ≥ 0.54

Univariate analyses also showed that older age (≥62 vs <62 years, *P* = 0.009), metastasis stage (metastasis vs locally advanced, *P* < 0.001), an elevated NLR (≥5 vs <5, *P* < 0.001), higher mGPS scores (1 + 2 vs 0, *P* < 0.001), and non-chemotherapeutic treatment (no chemotherapy vs chemotherapy, *P* < 0.001) were significantly associated with worse OS in advanced PC.

**Fig. 3:**
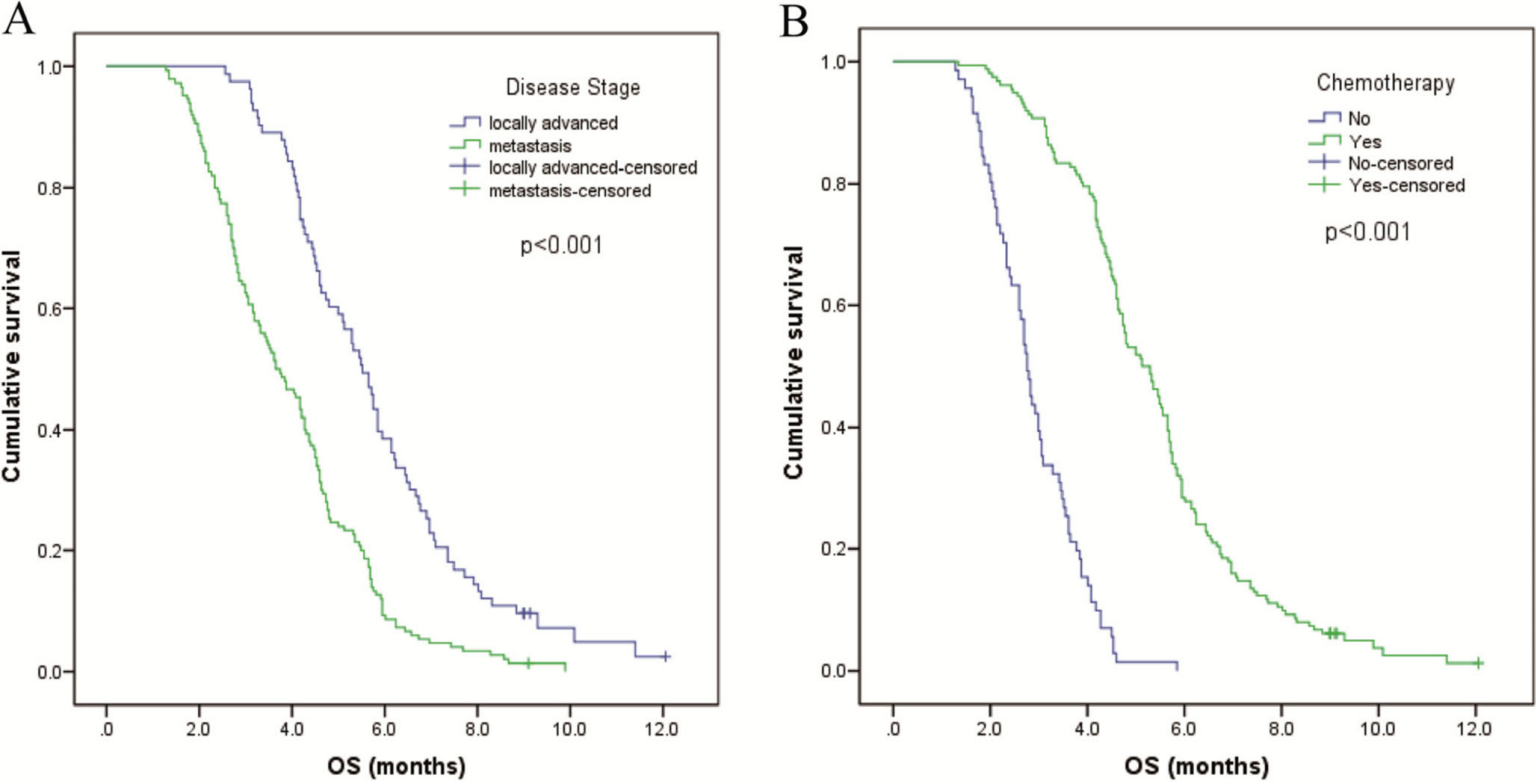
The overall survival curves according to **a** disease stage, and **b** treatment with or without chemotherapy

To investigate whether CRP/Alb ratio could serve as an independent prognostic factor in advanced PC, multivariate analyses were also conducted. In the multivariate analyses, patients in the higher CRP/Alb group had a worse outcome than those in the lower ratio group (hazard ratio (HR) 3.995; 95 % CI 2.644–6.034; *P* < 0.001) (Table 3). In addition, the age (*P* < 0.001), disease stage (*P* < 0.001), and chemotherapy (*P* < 0.001) independently and significantly predicted OS, whereas the NLR, PLR, and mGPS did not predict OS (Table 3 and Fig. 3). Moreover, subgroup analyses were also conducted. Although patients in the CRP/Alb-high group had a worse OS than the CRP/Alb-low group, chemotherapy still contributed to a significantly longer median survival in both groups (CRP/Alb < 0.54 group, 5.7 vs 3.0 months, *P* < 0.001; CRP/Alb ≥ 0.54 group, 3.7 vs 2.0 months, *P* < 0.001) (Fig. 3).

**Fig. 4:**
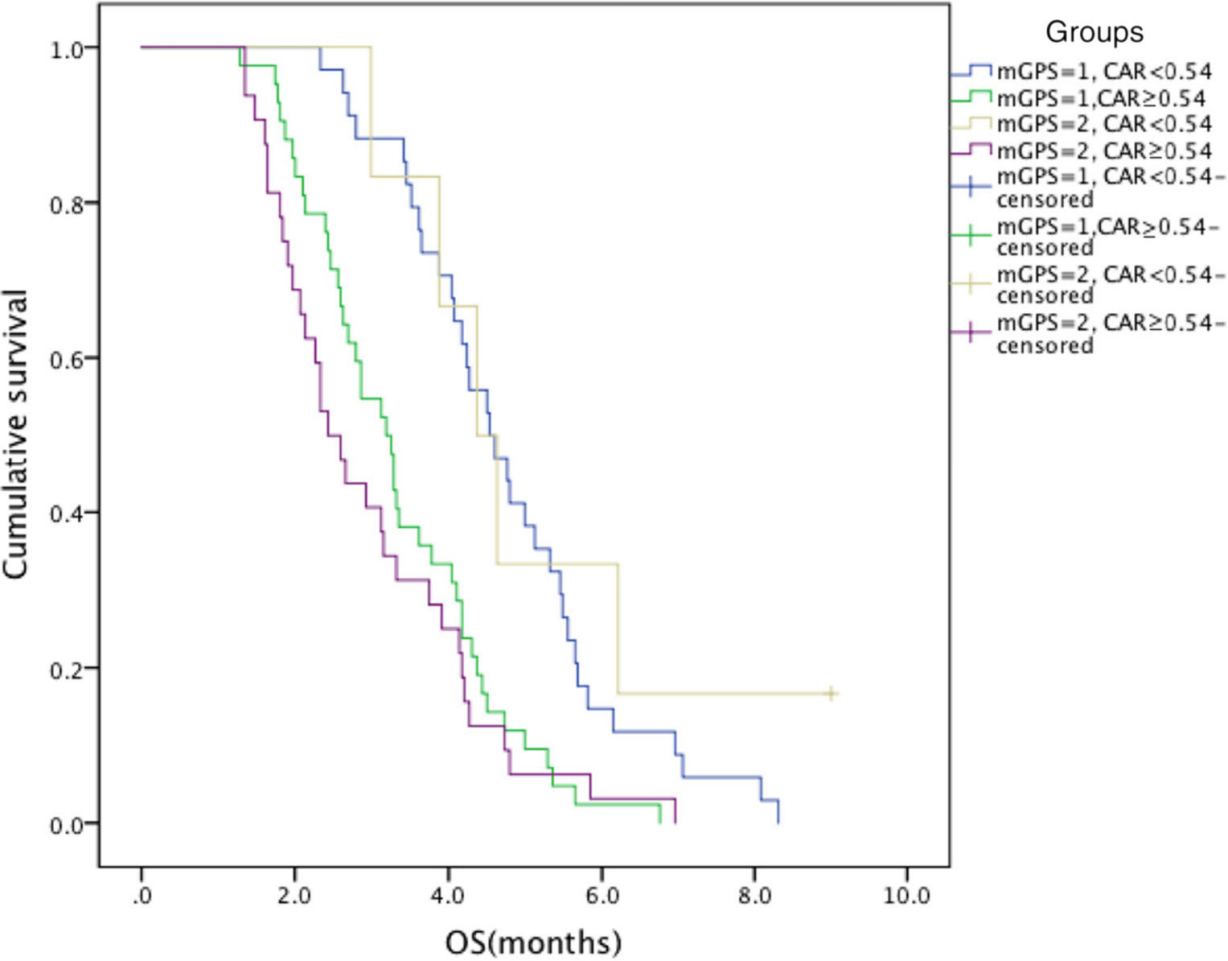
Kaplan–Meier curves between groups based on the mGPS scores and CRP/Alb ratio

To identify the interaction between the CRP/Alb ratio and mGPS for OS, we categorized patients into four groups based on two indexes, including mGPS score of 1 and CRP/Alb ratio < 0.54; mGPS score of 1 and CRP/Alb ratio ≥ 0.54; mGPS score of 2 and CRP/Alb ratio < 0.54; and mGPS score of 2 and CRP/Alb ratio ≥ 0.54. The survival analyses indicated that compared with patients who had an mGPS score of 1 and CRP/Alb ratio ≥ 0.54, those with an mGPS score of 1 and CRP/Alb ratio < 0.54 had a longer median OS (4.5 vs 3.2 months, *P* < 0.001), which was similar to the comparison of an mGPS score of 2 and CRP/Alb ratio < 0.54 with an mGPS score of 2 and CRP/Alb ratio ≥ 0.54 (4.4 vs 2.4 months, *P* = 0.023). However, no significant differences were found between an mGPS score of 1 and CRP/Alb ratio < 0.54 and an mGPS score of 2 and CRP/Alb ratio < 0.54 (4.5 vs 4.4 months, *P* = 0.370) or between an mGPS score of 1 and CRP/Alb ratio ≥ 0.54 and an mGPS score of 2 and CRP/Alb ratio ≥ 0.54 (3.2 vs 2.4 months, *P* = 0.416) (Fig. 4).

## Discussion

Despite recent improvements in the validated benefit of chemotherapy and spread of multidisciplinary therapy for patients in the advanced stage, pancreatic cancer remains a devastating disease with an extremely poor prognosis. Optimal responses will be achieved only if the treatment plan is tailored for individuals based on accurate and stable prediction of potential survival. However, tumor-related parameters such as the pathological stage and resectability of the primary tumor primarily play a role in evaluating disease and predicting the outcome.

Therefore, an increasing number of thorough studies have identified some easily available predictors for substituting the traditional surgical method. A diverse set of observations offers evidence supporting the correlation between inflammation and cancer. While early studies were devoted to elucidating the link between pre-existing inflammation and consequent tumor initiation, more recent studies tend to add new insight into tumor-associated inflammation and the host response to oncogenic change in the intrinsic pathway. For the intrinsic pathway, inflammation and tumor growth are driven by genetic mutations that cause the activation of transcription factors, release of inflammatory mediators (chemokines, cytokines, and prostaglandins), and subsequent infiltration of inflammatory cells and angiogenesis [5]. This inflammatory condition further promotes the malignant progression of tumors, enhances local immunosuppression, induces invasion and metastatic spread, and influences the treatment response [5, 22, 23].

Among the many inflammatory mediators, interleukin-6 (IL-6) is a notable inflammatory cytokine secreted by innate immune cells that regulates the levels of C-reactive protein (CRP), an acute-phase protein [24]. Recently, it was reported that elevated pretreatment serum IL-6 and CRP levels showed a significant relationship with a poor outcome [25–28]. Additionally, the serum albumin level is not only a reflection of nutritional state but also it is, to a larger degree, a consequence of the inflammatory state. Plausible explanations are as follows. When considered with tumor-related systematic inflammation, the ability of the liver to produce albumin decreases as a result of increased acute-phase protein synthesis [29, 30]. Alternatively, the release of cytokines from inflammatory cells increases the microvascular permeability, increasing the flow of serum albumin towards the extravascular compartment [31]. Therefore, the link between hypoalbuminemia and inflammation is also quite strong [32]. Meanwhile, the role of the pretreatment serum albumin levels as an independent predictor of the OS has been demonstrated in various cancers, including PC [33–35].

These achievements greatly piqued our interest in combining CRP and albumin into a noble potential inflammatory prognostic indicator in advanced PC and identifying its predictive value. In addition, some recent studies have demonstrated that the CRP/Alb ratio is a promising inflammation-associated prognostic factor in cancer, including liver, lung, gastric, and esophageal cancer [8, 13–17]. In our study, we assessed and compared the prognostic value of the NLR, PLR, mGPS, and CRP/Alb ratio in advanced PC by retrospectively analyzing the pretreatment laboratory data of 233 eligible patients. According to statistical analysis, there was a significant association between the CRP/Alb ratio and other inflammatory indexes (excluding PLR), which might suggest that comprehensive evaluation of these inflammatory parameters could provide a more advisable prognostic estimate. In accordance with the result of the chi-square test of the CRP/Alb ratio versus disease stage, the CRP/Alb ratio remained a significant prognostic parameter regardless of the stage of advanced PC in subgroup analyses. After excluding the confounding factors from the multivariate analyses, the CRP/Alb ratio remained the only significant inflammation-related prognostic index. To the best of our knowledge, this is the first study to explore the role of the CRP/Alb ratio as a predictor of prognosis in advanced PC.

As the mGPS and CRP/Alb ratio both include the CRP and albumin levels, comparison of the two factors was performed. Because the CRP/Alb ratios of patients with mGPS scores of 0 were all less than 0.54, dichotomizing patients with mGPS scores of 1 and 2 on the basis of the CRP/Alb ratio was conducted. Kaplan–Meier tests showed that patients with an mGPS score of 1 and CRP/Alb ratio < 0.54 and an mGPS score of 2 and CRP/Alb ratio < 0.54 had comparable longer OS. Similarly, those in the groups with an mGPS score of 1 and CRP/Alb ratio ≥ 0.54 and an mGPS score of 2 and CRP/Alb ratio ≥ 0.54 had parallel poor outcome. The results exposed defects in the mGPS prognostic ability for patients with scores of 1 and 2 because they did not have significant differences in OS; by contrast, they could be distinguished according to the CRP/Alb ratio. This disadvantage arises from its nature as a categorized variable that fails to accurately reflect the disease condition of every patient. In conclusion, the CRP/Alb ratio is superior to the other inflammation-related prognostic factors. Our findings may have practical value in the therapy of advanced PC patients. Patients with a high CRP/Alb ratio may require more active adjuvant chemotherapy.

The generalizability of the conclusions is limited by the threatened independence of the variables. As a retrospective and single-center study, the limitations of the current research lie in its intrinsic features. To narrow down the inevitable selection bias, we enrolled consecutive patients and included a relatively large sample size. Meanwhile, we explored the prognostic significance of the CRP/Alb ratio in a multi-faceted approach, including validation of the value at the level of all patients and patient subgroups based on disease stage. However, a multicenter prospective validation study with a larger scale sample is needed to confirm our findings.
